# High-affinity peptide ligand LXY30 for targeting α3β1 integrin in non-small cell lung cancer

**DOI:** 10.1186/s13045-019-0740-7

**Published:** 2019-06-10

**Authors:** Wenwu Xiao, Weijie Ma, Sixi Wei, Qianping Li, Ruiwu Liu, Randy P. Carney, Kevin Yang, Joyce Lee, Alan Nyugen, Ken Y. Yoneda, Kit S. Lam, Tianhong Li

**Affiliations:** 1 Department of Biochemistry and Molecular Medicine, University of California Davis, Sacramento, CA 95817 USA; 2 Division of Hematology/Oncology, Department of Internal Medicine, University of California Davis School of Medicine, University of California Davis Comprehensive Cancer Center, 4501 X Street, Suite 3016, Sacramento, CA 95817 USA; 3 Present Address: Department of Biochemistry, Hospital Affiliated to Guizhou Medical University, Guiyang, Guizhou China; 4 Present Address: Department of Cardiothoracic Surgery, Shanghai Jiaotong University Affiliated Sixth People’s Hospital, 600 Yi-Shan Road, Shanghai, 200233 China; 5 Department of Biomedical Engineering, University of California Davis, Davis, CA USA; 6 Present Address: Perelman School of Medicine, University of Pennsylvania, Philadelphia, PA USA; 7 Department of Pharmacy, University of California Davis Health System, Sacramento, CA 95817 USA; 8 Division of Pulmonary, Critical Care, and Sleep Medicine, Department of Internal Medicine, University of California Davis School of Medicine, Sacramento, CA USA; 9 Department of Internal Medicine, Veterans Affairs Northern California Health Care System, Mather, CA USA

**Keywords:** Cancer-targeting peptide, α3β1 integrin, Non-small cell lung cancer, Exosomes, In vivo imaging, Patient-derived xenograft

## Abstract

**Background:**

α3β1 integrin is a promising cancer biomarker and drug target. We previously identified a 9-amino-acid cyclic peptide LXY30 for detecting α3β1 integrin on the surface of live tumor cells. This study was undertaken to characterize LXY30 in the detection, cellular function, imaging, and targeted delivery of in vitro and in vivo non-small cell lung cancer (NSCLC) models.

**Methods:**

The whole-cell binding assay was performed by incubating NSCLC cells, extracellular vesicles (EVs), and peripheral blood mononuclear cells (PBMCs) with TentaGel resin beads coated with LXY30. In this study, we defined the nanosize EVs as exosomes, which were characterized by flow cytometry, transmission electron microscopy, dynamic light scattering, and Western blots. The function of LXY30 was determined by modulating the epidermal growth factor receptor (EGFR) signaling pathway by growth inhibition and Western blots. For in vivo biodistribution, mice bearing subcutaneous and intracranial NSCLC xenograft tumors were administrated intraveneously with LXY30-biotin/streptavidin-Cy5.5 complex and then analyzed for in vivo and ex vivo optical imaging and histopathology.

**Results:**

We showed that LXY30 specifically and sensitively detected α3β1 integrin-expressing NSCLC cells and tumor-derived exosomes. Tumor DNA isolated from LXY30-enriched plasma exosomes might be used to detect driver oncogenic mutations in patients with metastatic NSCLC. LXY30 only enriches tumor cells but not neutrophils, macrophages, or monocytes in the malignant pleural effusion of NSCLC patients for detecting genomic alterations by next-generation sequencing. LXY30 detected increased α3β1 integrin expression on the *EGFR*-mutant NSCLC cells with acquired resistance to erlotinib compared to parental erlotinib-sensitive *EGFR*-mutant NSCLC cells. We further showed that LXY30 modulated the EGFR signaling pathway independently from another peptide ligand LXW64 targeting αvβ3 integrin in erlotinib-resistant, *EGFR*-mutant H1975 cells. Analysis of The Cancer Genome Atlas (TCGA) revealed high α3 integrin expression was associated with poor prognosis in lung squamous cell carcinoma. LXY30-biotin/streptavidin-Cy5.5 complex had higher uptakes in the subcutaneous and intracranial xenografts of various α3β1 integrin-expressing lung adenocarcinoma and patient-derived lung squamous cell carcinoma xenografts while sparing the surrounding normal tissues.

**Conclusion:**

LXY30 is a promising peptide for the cancer diagnosis and in vivo targeted delivery of imaging agents and cancer drugs in NSCLC, independent of histology and tumor genotype.

**Electronic supplementary material:**

The online version of this article (10.1186/s13045-019-0740-7) contains supplementary material, which is available to authorized users.

## Background

Non-small cell lung cancer (NSCLC) is the most common and leading cause of cancer-related death in the USA and worldwide [1, 2]. With the rapid advances in our understanding of tumor biology and genomics technology, NSCLC has been recognized as a molecularly and genomically complex and heterogeneous disease [3]. Despite advances in early detection and treatment, far too many patients present with locally advanced or metastatic NSCLC and their prognosis remains poor [4]. Novel targets and treatment strategies are needed to further improve the clinical outcomes for NSCLC patients.

Integrins have been explored as novel cancer biomarkers and drug targets. Integrins are 24 heterodimeric cell surface receptors that mediate cell adhesion, signaling transduction, tumorigenesis, and metastasis [5, 6]. While the integrin α unit cooperates with β unit to mediate the binding to various ligands and substrates, the integrin β unit mainly mediates the complex biological functions. Each integrin has distinct cellular distributions and mediates distinct biological functions. Abnormal integrin expression has been reported in many cancer types including NSCLC [7]. Integrins do not possess intracellular tyrosine kinase domains and rely on the receptor tyrosine kinases (RTKs) of associated signaling molecules, such as fibroblast growth factor receptor (FGFR) or epidermal growth factor receptor (EGFR), for their function [8, 9]. Analysis of the lung adenocarcinoma metastasis network identified α3β1 integrin as the surface receptor that mediates adhesion and seeding in vitro and in vivo of lung adenocarcinoma [10]. α3β1 integrin is a heterodimeric receptor for fibronectin, laminin, collagen, epiligrin, thrombospondin, and chondroitin sulfate proteoglycan 4 (CSPG4). Cross talk between RTK and integrin is synergistic for survival (PI3K, AKT, and NFκB), adhesion (FAK and integrin function), and growth/motility (RTK and downstream pathways including ERK 1 and 2) [11]. Overexpression of α3β1 integrin has been detected in multiple tumor types and associated with poor prognosis, tumorigenesis, invasion, metastasis, and resistance to cancer treatment in several cancer types, including NSCLC [12–15].

No cancer therapy targeting α3β1 integrin is in clinical evaluation. Targeting the extracellular domain of integrins has not been proven an effective anti-cancer therapeutic strategy, largely because natural integrin ligands have low affinity for binding to tumor cells and do not significantly alter the biological properties of tumor cells. We previously generated and characterized several peptide ligands for integrins expressed on live tumor cells using the invented one-bead-one-compound combinational chemistry library approach [16–19]. Among them, LXY30 was bound to α3 integrin on the surface of a panel of NSCLC cell lines with variable affinities [20]. As integrin α3 subunit only forms a heterodimer with the integrin β1 subunit, LXY30 is a promising peptide ligand for in vivo targeting α3β1 integrin in NSCLC. The objective of this study was to characterize the role of LXY30 in the diagnosis, imaging, and targeted drug delivery using various in vitro and in vivo NSCLC models.

## Materials and methods

### Synthesis of peptides, peptide-FITC, and peptide-biotin conjugates

Peptide-biotin and peptide-fluorescein isothiocyanate (FITC) were designed by attaching biotin or FITC to the side chain of Lys and two hydrophilic linkers between peptide and Lys (biotin) and Lys (FITC) on Rink amide MBHA resin as previously described [20]. Standard solid-phase peptide synthesis approach using Fmoc/tBu chemistry and 6-chloro-*N*-hydroxybenzotriazole (6-Cl HOBt)/1,3-diisopropylcarbodiimide (DIC) coupling was employed to synthesize the linear peptides and peptide conjugates on Rink amide resin (loading 0.52 mmol/g) (GL Biochem, Shanghai, China) as described [17, 19, 21]. The disulfide formation was achieved using CLEAR-OX^TM^ resin (Peptide International Inc, Louisville, KY) in 50% of 0.1 M ammonium acetate buffer in acetonitrile (ACN). The collected liquid was lyophilized to yield crude products that were purified by reversed-phase HPLC with a purity of > 95%. The identities of compounds were confirmed with matrix-assisted laser desorption/ionization time of flight mass spectrometry (MALDI-TOF MS). Analytical HPLC was performed on a Waters 2996 HPLC system equipped with a 4.6 × 150 mm Waters Xterra MS C18 5.0 μm column and employed a 20-min gradient from 100% aqueous H_2_O (0.1% trifluoroacetic acid, TFA) to 100% ACN (0.1% TFA) at a flow rate of 1.0 mL/min. Preparative HPLC was performed on a System Gold 126NMP solvent module (Beckman) with a C18 column (Vydac, 10 μm, 2.2 cm i.d. × 25 cm). A gradient elution of 0–60% B over 45 min, then 60–100% B over 5 min, and followed by 100% B for 5 min was used at a flow rate of 5 mL/min (solvent A, H_2_O/0.1% TFA; B, acetonitrile/0.1% TFA).

### Human NSCLC models

Human NSCLC cell lines H1975 and A549 were obtained from American Type Culture Collection (Manassas, VA). H3255 was a gift from Dr. Pasi A. Janne (Dana-Farber Cancer Institute, Boston, MA). H3255 R#2 is the best-characterized resistant clone mimicking the acquired resistance model of *EGFR*-mutant NSCLC to first-generation EGFR TKI erlotinib [22]. Patient biospecimens were collected under an institutional review board (IRB)-approved protocol (Protocol No. 226210) at the University of California, Davis.

### Isolation of exosomes from tumor cells or patient’s malignant pleural effusion

Our group recently isolated and comprehensively characterized LXY30-enriched, nanosized extracellular vesicles (EVs) from ovarian tumor cells with a composition reflecting the cells’ biological state [23]. The collected EVs were confirmed as exosomes according to the International Society for Extracellular Vesicles (ISEV) suggested standards [24, 25]. Standard differential centrifugation protocols were used to isolate exosomes from both cancer cell culture supernatant and pleural effusion [26, 27]. Tumor cells were cultured in exosome-free medium in T75-cm^2^ flasks for 3 days until they reached a confluency of 70–80%. The media or supernatant from patient’s pleural effusion specimens was collected and centrifuged at 2,000*g* for 20 min followed by 10,000*g* for 30 min to remove the cellular debris. The resulting media or supernatant samples were filtered through a 0.22-µM filter (Millipore, Boston, MA), followed by being ultrafiltered through Amicon® Ultra 15 mL Centrifugal Filters (Millipore, Boston, MA) to enrich the exosomes. For the purification of circulating EVs from patients, we used a commercial exosome isolation kit, and exosome-enriched media were combined with 1/2 volume of Total Exosome Isolation Reagent (Thermo Fisher Scientific, Waltham, MA) and mixed well by vortexing or pipetting up and down until a homogenous solution was formed. The resulting solution was incubated at 4 °C overnight and centrifuged at 4 °C at 12,000×*g* for 1 h. The supernatant was discarded, and the purified EVs were resuspended in about 500 μL 1X PBS buffer and stored at − 80 °C until further analysis. These EVs were confirmed to be enriched in “exosome” type via flow cytometry, transmission electron microscopy (TEM) or nanoparticle tracking analysis (NTA), dynamic light scattering (DLS), and Western blots.

### On-bead whole-cell binding assay

Tumor cells from human NSCLC cell lines, patient’s malignant pleural effusion, or PBMCs from patients with advanced NSCLC were collected, spun down, and resuspended in 10 mL of culture medium in a 10-cm Petri dish. For the whole-cell binding assay, 5 μL of beads coated with a known peptide sequence was washed sequentially with ethanol, water, and PBS. The beads were then incubated with suspended cells in the dish, and the entire dish was swirled at a speed of 40 rpm in an incubator at 37 °C and 5% CO_2_. The plate was then examined under an inverted microscope every 15 min to check the cell binding. To determine the binding sensitivity of LXY30, A549 cells or malignant pleural effusion (PE) was subjected to a serial dilution (1:10^5^ or 1:10^3^, respectively) using 1 mL of supernatant of malignant pleural effusion from NSCLC patients, followed by incubation with ~ 250 TentaGel (90 μm, 0.26 mmol/g) (Rapp Polymere GmbH, Tϋbingen, Germany) beads coated with LXY30 or scrambled-LXY30 (S-LXY30) for 2 h before examination under microscope.

### Exosome-bead binding assay and confocal microscopy

For the exosome-bead binding assay, 1.5 μg/μL A549, H1975, or patient tumor-derived exosomes in 200 μL were added into 1.5 mL tube followed by 100 TentaGel beads coated with LXY30 or S-LXY30 at 37 °C for 60 min, respectively. The exosome-beads were then washed three times in PBS. After the wash, Alexa Fluor® 647 mouse anti-human CD63 antibody (Biolegend, San Diego, CA) was added into the tube, incubating for 1 h and then washed three times in PBS. Next, A549 exosome-bead and H1975 exosome-bead binding were visualized under a LSM710 confocal fluorescence microscope (Zeiss, Germany).

### Flow cytometry

Confluent (70–80%) human NSCLC cell lines and tumor cells isolated from patient pleural effusion were dissociated with 0.05% trypsin-EDTA and neutralized with culture medium. PBMCs were directly collected from the blood via Ficoll-Paque density gradient centrifugation. Each sample contained 3 × 10^5^ cells and was incubated with biotinylated peptides in 50 μL of PBS containing 10% FBS and 1 mM MnCl_2_ for 30 min on ice. Each sample was washed three times with 1 mL of 1X PBS containing 1% FBS and incubated with a 1:500 dilution of streptavidin-PE (1 mg/mL) for 30 min on ice followed by a single wash with 1 mL of PBS containing 1% FBS. The final samples were analyzed by flow cytometry (Becton Dickinson Fortessa Flow Cytometer, San Jose, CA). Histogram analysis with mean fluorescence intensity (MFI) was analyzed using FlowJo 7.6.1 program (Ashland, OR).

### Analysis of cellular proliferation and function by cell attachment assay and Western blotting analysis

Six-well plates were coated with 1500 μL of 20 μg/mL avidin (Thermo Fisher Scientific) and incubated for 1 h at 37 °C. Avidin-coated wells were washed three times with PBS and incubated with 1500 μL molar equivalents (2 μM) of D biotin (Thermo Fisher Scientific), LXY30-biotin, LXW64-biotin, or LXY30- and LXW64-biotin combo for 1 h. The wells were washed three times with PBS and blocked with 1% BSA (Thermo Fisher Scientific) for 1 h. After the wells were washed three times with PBS, 1 × 10^5^ H1975 cells were suspended in the maintenance medium, added to the wells, and incubated for 72 h at 37 °C, 5% CO_2_. On the indicated time point (8 h, 24 h, 48 h, 72 h), trypsinized cells were counted by using hemocytometer for cell number analysis. After incubation for 72 h, one million cells were lysed in the Radio-Immunoprecipitation Assay buffer (Thermo Fisher, Waltham, MA). Thirty micrograms of lysates or exosome samples was separated by electrophoresis on 10% SDS-PAGE gels, transferred to nitrocellulose membranes, and probed with the following primary antibodies at 1:400 dilution: pEGFR Y1068, EGFR, pAKT S473, AKT (40D4), pMEK1/2 S217/221, MEK1/2 47E6, pSTAT3 Y705, and STAT3 124H6 (all from Cell Signaling Technology, Danvers, MA); CD63 (Santa sc-365604), integrin α3 (sc-374242), integrin β1 (sc-59829), integrin αV (sc-9969), and β-actin (sc-47778) (Santa Cruz Biotech). The secondary antibody was anti-mouse IgG, HRP-linked antibody (cell signaling, 1:500; #7076) or anti-rabbit IgG, HRP-linked antibody (cell signaling, 1:500; #7074). Densitometry was performed with Gel Doc^TM^ software (XR+ Imager, Bio-Rad, USA). The expression of each protein was normalized to β-actin in each sample.

### Tumor xenografts

Mice studies were performed according to an Institutional Animal Care and Use Committee (IACUC)-approved protocol (Protocol No. 20080) at the University of California, Davis. Female athymic nude mice (nu/nu), obtained from Harlan (Indianapolis, IN) at 5–6 weeks of age, were injected with 5 × 10^6^ of H3255, H1975, or A549 cells subcutaneously in the right flank. Patient-derived xenograft (PDX) model was generated from a patient with metastatic squamous cell lung cancer [28] implanted into the flank of NSG mice at age of 5–6 weeks old. For the intracranial implantation, 2.5 × 10^5^ cells in 5 μL PBS were injected into the right striatum area of the mouse with the aid of a mouse stereotactic instrument (Stoelting Co, Wood Dale, IL, USA). When the subcutaneous tumors reached 0.5–1.0 cm in diameter or 21–28 days after implantation, the mean size of intracranial xenograft tumors was 0.2–0.5 cm in diameter. Mice bearing NSCLC tumors were subjected to in vivo and ex vivo imaging studies.

### In vivo and ex vivo optical imaging

Biotinylated peptide-SA-Cy5.5 (1.8 nmol), prepared by mixing 7.2 nmol of biotinylated peptide with 1.8 nmol of streptavidin-Cy5.5 in PBS overnight at 4 °C, was injected via the tail vein in an anesthetized mouse before imaging. Animals were placed in the supine, prone, or lateral position. Images were acquired by a Kodak IS2000MM image station (Rochester, NY) with a 625/20 band-pass excitation filter, 700WA/35 band-pass emission filter, and 150 W quartz. Halogen lamp light source was set to maximum. Images were captured at different time points with a CCD camera set at F stop = 0, FOV = 150, and FP = 0. Mean fluorescence intensity (MFI) was calculated by drawing the region of interest (ROI) of the mouse tumor using the Kodak ID 3.6 software. For ex vivo imaging, the mice were euthanized, and their organs were excised for imaging.

### H&E staining

Cryosections of tumor xenografts in 10 μm thickness were fixed with 4% paraformadehyde for 20 min. After rinsing with deionized water, the slides were stained with hematoxylin for 5 s, rinsed in tap water, dipped in eosin for 30 s, and then dehydrated for 5 s with 95% ethanol, 5 s with 100% ethanol, and 15 s with xylene. The slides were covered with Permount solution and coverslips and examined with fluorescence microscope (IX81; Olympus) (image software: Metaphore).

### In vitro fluorescence and confocal microscopy

For assessing the expression of targeted integrin in the xenograft tumors, 10-μm-thick slides of xenograft tumors were stained with mouse anti-α3 integrin antibody at 1:200 for 2 h. After washing with PBS, the slides were incubated with chicken anti-mouse Alexa 594 (Thermo Fisher Scientific, Waltham, MA) at 1:1000 for 30 min and washed with PBS. The slides were then covered by mounting solution with DAPI and evaluated under a fluorescence microscope (IX81; Olympus) (image software: Metaphore). For the cell line uptake experiment, A549 cells adhering on the bottom of chamber slides were incubated with 1 μM biotinylated LXY30 streptavidin-Alexa 594 conjugations for 2 h then observed under confocal fluorescence microscope (LSM710; Zeiss). For the H3255 intracranial xenograft microscopy, 10 μM cryosections of intracranial H3255 tumor after injection with LXY30-biotin/streptavidin-Cy5.5 complex were fixed in acetone at − 20 °C for 20 min. After washing with PBS, the sections were mounted and observed under the fluorescence microscope.

### Data and statistical analyses

Descriptive statistics for continuous and categorical variables were stratified by binding to each integrin subtype or marker. All data are shown as mean ± standard deviation (SD) with at least 3 independent measurements. The two-sample *t* test was used for continuous variables. All analyses were conducted using SAS, university edition 2.5 9.4 M4 (SAS Institute, Cary, NC), and figures were made using GraphPrism software (Version 7.03). All statistical tests were two sided, and a *p* value less than 0.05 was considered statistically significant.

### Data availability

The datasets generated and/or analyzed during this study, as well as the computer code used to perform statistical analysis, are available from the corresponding authors on reasonable request. Supplementary information about IHC procedures, exome sequencing, and generation of exome-derived variables are available in supplementary methods.

## Results

### LXY30 specifically and sensitively detected α3 integrin-expressing NSCLC cells and tumor-derived exosomes

**Fig. 1:**
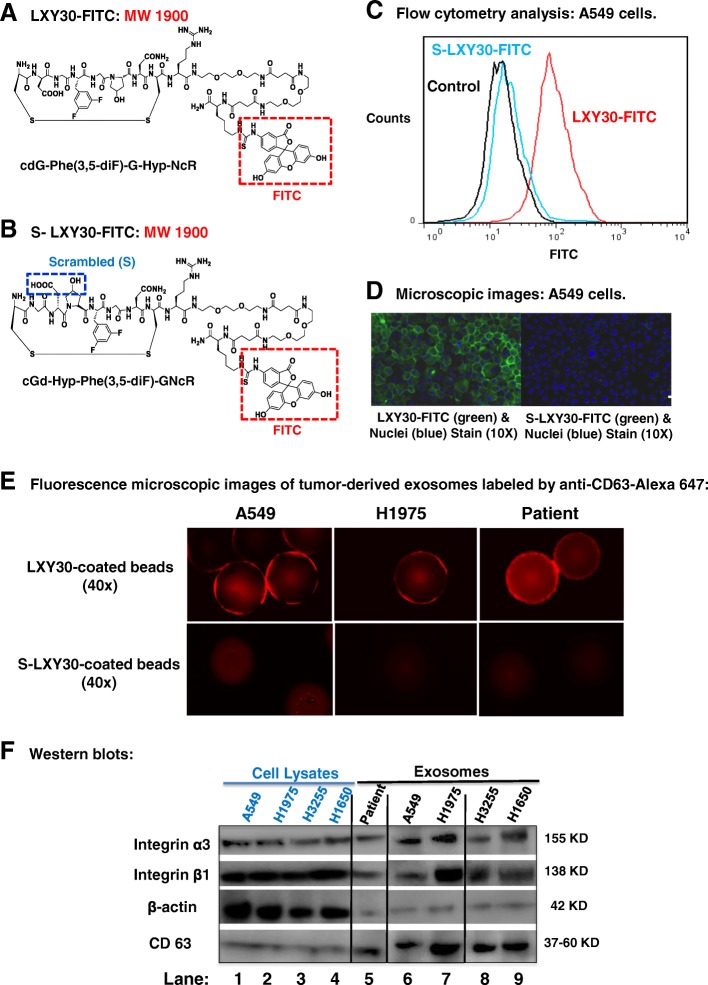
LXY30 specifically binds to integrin α3β1-expressing NSCLC cells and their derived exosomes. Chemical structures of LXY30-FITC (**a**) and scrambled-LXY30 (S-LXY30)-FITC (**b**) are illustrated. A549 cells growing in DMEM medium were collected and incubated with LXY30-FITC or S-LXY30-FITC for 2 h, and the binding specificity of LXY30 was evaluated by flow cytometry (**c**) and fluorescence microscopy (**d**). Exosomes were isolated from the supernatant of two integrin α3β1-expressing NSCLC cells (A549 and H1975) and a patient’s malignant pericardial effusion (**e**). Exosomes were incubated with TentaGel bead coated with LXY30 (left panel) or S-LXY30 (right panel) for 2 h, followed by incubation with Alexa 647 mouse anti-CD63 antibody (red) for 1 h before being visualized by fluorescence microscopy (**e**). The expression of integrin α3, integrin β1, and exosomal marker CD63 in 4 NSCLC cell lines and one patient pericardial effusion were determined by Western blots (**f**). Beta-actin was used as loading control for cellular protein expression. The protein loading for cell lysate is around 5 times lower than that of exosomes. h, hour(s); FITC, fluorescein isothiocyanate; NSCLC, non-small cell lung carcinoma

**Fig. 2:**
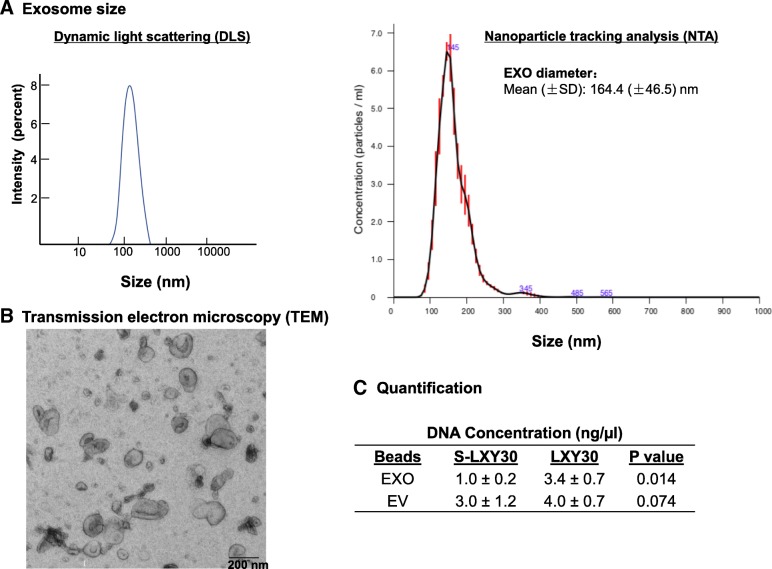
Characterization of tumor derived exosomes and EVs. The size and morphology of exosomes were evaluated by dynamic light scattering (DLS) and nanoparticle tracking analysis (NTA) (**a**) and transmission electron microscopy (TEM) (**b**), respectively. The yield of DNA was 3.4-fold higher in LXY30-enriched exosomes than in S-LXY30-enriched exosomes (3.4 ± 0.7 vs 1.0 ± 0.2 ng/μL, *p* = 0.014) (**c**). Driven mutations (EGFR L858R and T790 M in H1975) were detected by PCR and Sanger sequencing in the DNA isolated from LXY30 exosomes (**d**)

We previously reported that LXY30 binds to the α3 integrin on the surface of a panel of live NSCLC cell lines with variable affinities and entered the cells via endocytosis [20]. We further characterized the binding specificity of LXY30 to a representative α3 integrin-expressing A549 cells. As illustrated in Fig. 1, A549 cells are specifically bound to LXY30-FITC (Fig. 1a) but not to S-LXY30-FITC (Fig. 1b) as assessed by flow cytometry (Fig. 1c) and fluorescence microscopy (Fig. 1d). LXY30-FITC conjugate detected α3 integrin expression in 7 out of 8 (87.5%) NSCLC tumor cells isolated from patient’s biofluids (malignant pleural or pericardial effusion or plasma) (Table 1). The sensitivity was tested by serial dilutions of A549 cells, and LXY30 could detect individual A549 cells in 10^5^ dilutions (data not shown). We further showed that LXY30 but not S-LXY30 is specifically bound to α3 integrin-expressing exosomes from the supernatant of NSCLC cell lines or patient’s biofluids (malignant pleural or pericardial effusion or plasma) in 9 out of 10 (90%) patients (Table 1). Representative fluorescence images from the supernatant of 2 NSCLC cell lines (A549 and H1975) and a patient’s malignant pleural effusion are shown in Fig. 1e. The expression of α3 and β1 integrin subunits was found in exosomes isolated from the supernatant of 4 lung adenocarcinoma cell lines A549, H1975, H3255, and H1650 and a patient pericardial effusion by Western blots using exosomal (CD63) and cellular-specific (β-actin) markers (Fig. 1f). The size and morphology of exosomes (EXO) as nanosize EVs were confirmed by dynamic light scattering (DLS) and nanoparticle tracking analysis (NTA) (Fig. 2a) and transmission electron microscopy (TEM) (Fig. 2b), respectively. We analyzed the yield of DNA isolated from the EV and EXO (Fig. 2c). While there was no significant difference between the yield of DNA isolated from S-LXY30-enriched and LXY30-enriched EVs (4.0 ± 0.7 vs 3.0 ± 1.2 ng/μL, *p* = 0.074), the yield of DNA was 3.4-fold higher in LXY30-enriched exosomes than in S-LXY30-enriched exosomes (3.4 ± 0.7 vs 1.0 ± 0.2 ng/μL, *p* = 0.014). The same driven mutations (KRAS G12S in A549, EGFR L858R and T790 M in H1975) were detected in the DNA isolated from LXY30-enriched exosomes and tumor cells by polymerase chain reaction (PCR) and Sanger sequencing (Fig. 2d). Using 10 patient samples with known plasma EGFR mutations by a clinical next-generation sequencing assay, we could detect the same EGFR mutations in LXY30-enriched exosomes using Sanger sequencing when the mutation allelic frequency (MAF) was high (> 50%) but not low (< 10%) although the threshold was not determined.

**Table 1:** Integrin expression in tumor cells and tumor-derived exosomes isolated from NSCLC patients

Patient no.	Histology	LXY30 binding to tumor cells	LXY30 binding to exosomes	LXW64 binding to exosomes
1	LUSC	++	+	++
2	LUAD	+	+	+
3	LUAD	++	+	+
4	LUAD	+	NA	+
5	LUAD	+	+	−
6	LUAD	+	+	+
7	LUAD	−	−	−
8	LUAD	NA	+	NA
9	LUAD	NA	+	NA
10	LUAD	NA	+	NA
11	LUSC	+	+	−
Expression rate		87.5%	90%	62.5%
A549	LUAD	++	++	+
H1975	LUAD	++	++	+
H3255	LUAD	+	+	+
H1650	LUAD	++	+	+

### LXY30 specifically and sensitively detects live, circulating tumor cells in patient’s pleural effusion

**Fig. 3:**
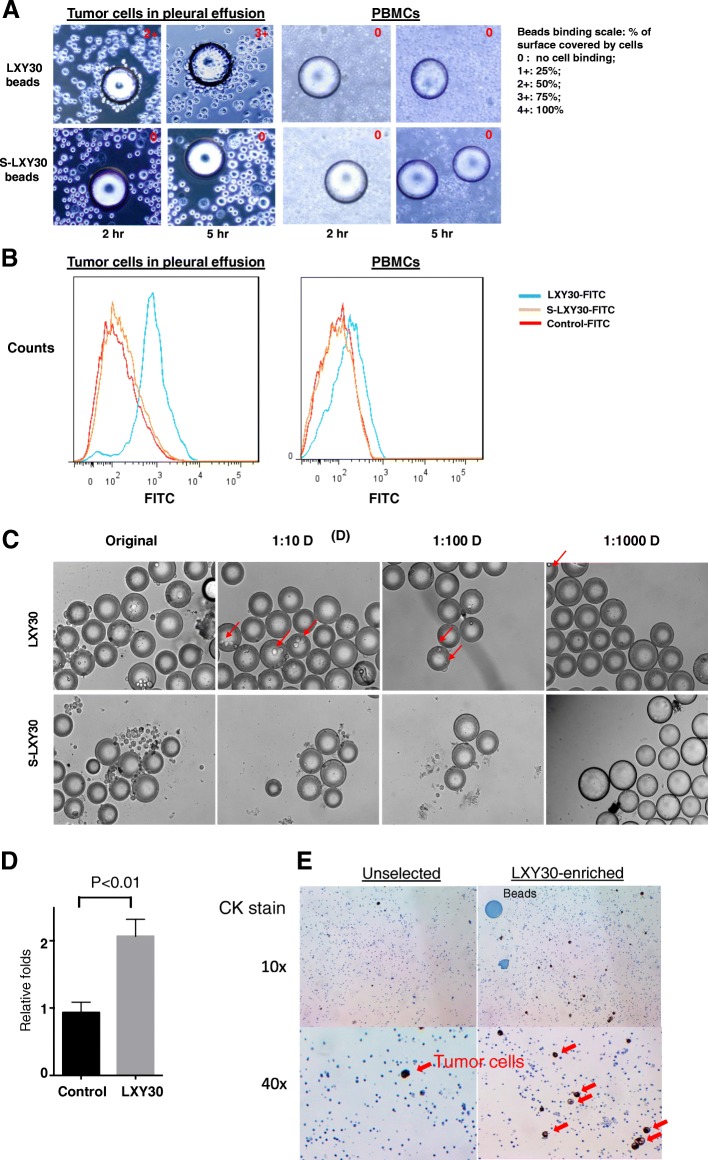
LXY30 specifically binds to live, circulating tumor cells in patient’s malignant pleural effusion. Representative tumor cells isolated from malignant pleural effusion from a patient with lung adenocarcinoma using Ficoll-Paque gradient separation bound to LXY30-coated but not S-LXY30-coated TentaGel beads after 2 h incubation (**a**, left panel). PBMCs isolated from the same patients did not bind to LXY30-coated or S-LXY30-coated beads (**a**, right panel). Flow cytometry confirmed that LXY30 specifically bound to tumor cells in malignant pleural effusion but not the PBMCs isolated from the same patient (**b**). Tumor cells from malignant pleural effusion of a newly diagnosed lung cancer patient cells were subjected to a serial dilution (1:10^3^) using 1 mL of supernatant, followed by incubation with ~ 250 TentaGel beads coated with LXY30 or S-LXY30 for 2 h. Representative images were visualized by microscope (× 10) (**c**). Arrow in red points to the individual live, bound tumor cells on the beads. On average, LXY30 enriched tumor cells by ~ 2-fold (**d**). Representative images showing tumor cells from malignant pleural effusion of a newly diagnosed lung cancer patient were visualized by light microscope (**e**). Red arrows point to the individual live tumor cells bound on the LXY30 beads (upper panel) but not S-LXY30 beads (lower panel). PBMC, pleural blood mononuclear cell

We further detected circulating tumor cells using LXY30 in 7 out of 8 (87.5%) patient’s pleural effusion. Figure 3 illustrates that representative tumor cells are isolated from malignant pleural effusion from a NSCLC patient using TentaGel beads coated with LXY30 but not S-LXY30 after 2- and 5-h incubation (Fig. 3a, left panel). LXY30 did not bind the inflammatory cells in the pleural effusion, such as macrophage and monocytes. Also, peripheral mononuclear blood cells (PBMCs) isolated from the same patients (*N* = 8) did not bind to TentaGel beads coated with either LXY30 or S-LXY30 (Fig. 3a, right panel). The specificity of binding was further confirmed by flow cytometry, in which FITC-labeled LXY30 specifically bound to tumor cells present in malignant pleural effusion but not the PBMCs in the blood of the same patient (Fig. 3b). We further showed that LXY30 sensitively detected individual, live pleural tumor cells in 1:1000 dilution using the supernatant from a patient’s malignant pleural effusion (Fig. 3c). On average, LXY30 could enriched malignant tumor cells by twofolds in these 8 patients tested (Fig. 3d). In a patient whose tumor cells were present in ~ 5% of cells in cell block, LXY30-bound beads were able to enrich the cellularity of tumor cells to > 20% in cell block (Fig. 3e), which enabled the successful detection of genomic alterations by next-generation sequencing assay.

### Increased expression of multiple integrins was observed in EGFR-mutant NSCLC cells gaining acquired resistance to erlotinib treatment

**Fig. 4:**
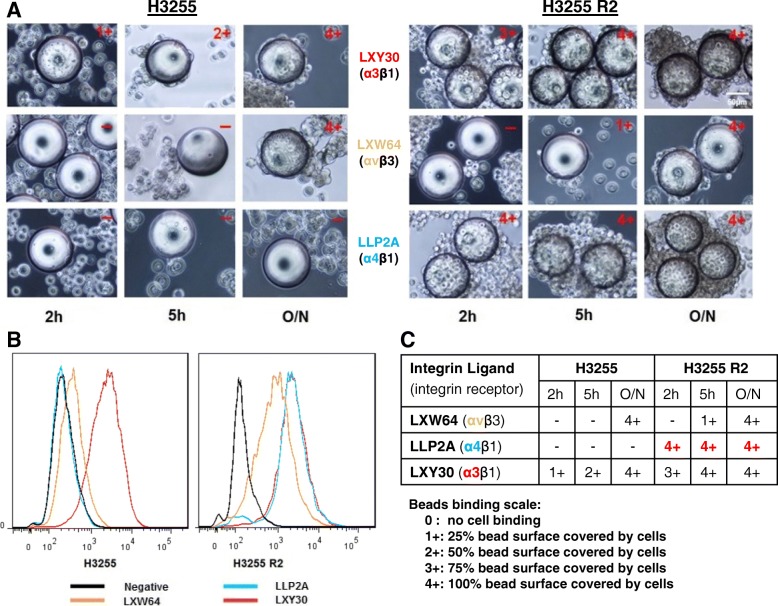
Erlotinib-resistant R#2 cells have increased integrin ligands binding compared to their parental erlotinib-sensitive, *EGFR-mutant* H3255 cells. H3255 (EGFR L585R) and its derived erlotinib-resistant R#2 cells were incubated with different integrin ligands (LXY30, LXW64, and LLP2A) for 2 h, 5 h, or overnight. The binding of tumor cells to integrin ligand-coated beads was visualized by light microscopy, and the intensity of binding was scored from negative (−), weakly, to strongly positive (1+ to 4+) (**a**). The binding of integrin ligands against tumor cells was confirmed by flow cytometry (**b**) and summarized in the table (**c**). h, hour(s); O/N, overnight; EGFR, epidermal growth factor receptor

A previous report suggests increased expression of multiple integrin subtypes in metastatic tumor cells compared to those of normal cells [29]. We further determined the integrin expression using the three most potent integrin ligands available (Additional file 1: Figure S1) on the surface of the EGFR-mutant NSCLC cells. Figure 4a illustrates that EGFR-mutant H3255 cells are bound to integrin ligand LXY30 (for α3β1 integrin) and LXW64 (for αvβ3 integrin) with high affinity but not to LLP2A (for α4β1 integrin) using the whole-cell binding assay. Consistent with previous reports [30, 31], we observed that erlotinib-resistant H3255 R#2 cells have increased the expression of all these three integrins compared to their parental erlotinib-sensitive, EGFR-mutant H3255 cells. The binding specificity of these cells to different peptide ligands was further confirmed by flow cytometry (Fig. 4b) and semi-quantified (Fig. 4c). As LLP2A could also bind to activated lymphocytes in PBMCs from cancer patients in clinical remission (data not shown), we further characterized the function of LXY30 and LXW64 in NSCLC in this study.

### LXY30 and LXW64 independently modulated different signaling molecules downstream of EGFR

**Fig. 5:**
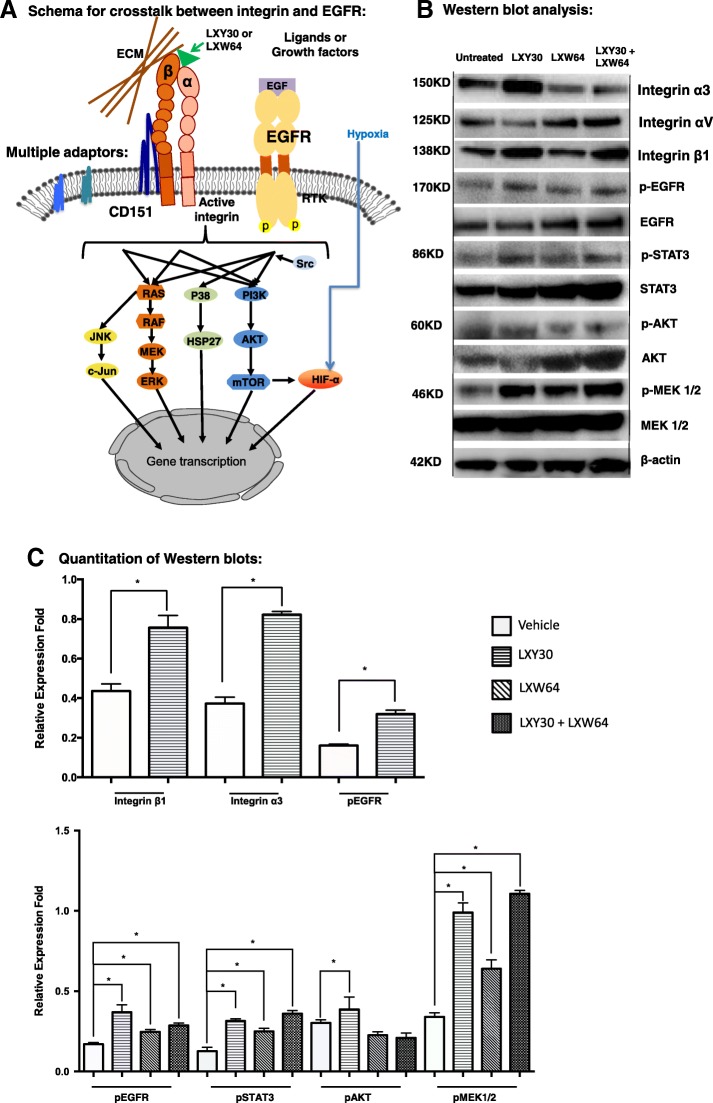
LXY30 activates EGFR and its downstream signaling molecules independently from another integrin ligand LXW64. Schematic representation for the cross talk between integrin and EGFR is illustrated (**a**). The effect of LXY30 and/or LXW64 on the expression of integrin subtypes, EGFR, and its key downstream signaling molecules in H1975 (*EGFR* L858R/T790 M) cells was analyzed after incubating with LXY30-biotin, LXW64-biotin, or LXY30-biotin/LXW64-biotin for 72 h by Western blot (**b**) and subsequently quantified (**c**). *p < 0.01

Integrins and EGFR share common downstream signaling pathways mediating the lung cancer initiation, growth, metastasis, and survival [32, 33]. LXY30 and LXW64 bind to the α3β1 and αvβ3 integrins, respectively, and activate EGFR and downstream signaling molecules (Fig. 5a). Compared to the untreated cells, LXY30 and LXY64 bind to erlotinib-resistant H1975 cells and activate the expression of α3, β1, and αv integrins; EGFR; and downstream signaling molecules either independently or in concert (Fig. 5b, c). The effect of LXY30 and/or LXW64 on the growth and proliferation of H1975 cells is shown by morphology (Additional file 2: Figure S2A) and cell counts (Additional file 2: Figure S2B) at 8 h, 48 h, and 72 h. Studies on other NSCLC cell lines revealed that the effect of LXY30 and LXW64 might be cell line dependent (Table 1), although the underlying mechanisms remain to be defined.

### In vivo targeting of LXY30 to both subcutaneous and intracranial xenograft tumors of integrin α3β1-expressing EGFR-mutant H3255 cells

**Fig. 6:**
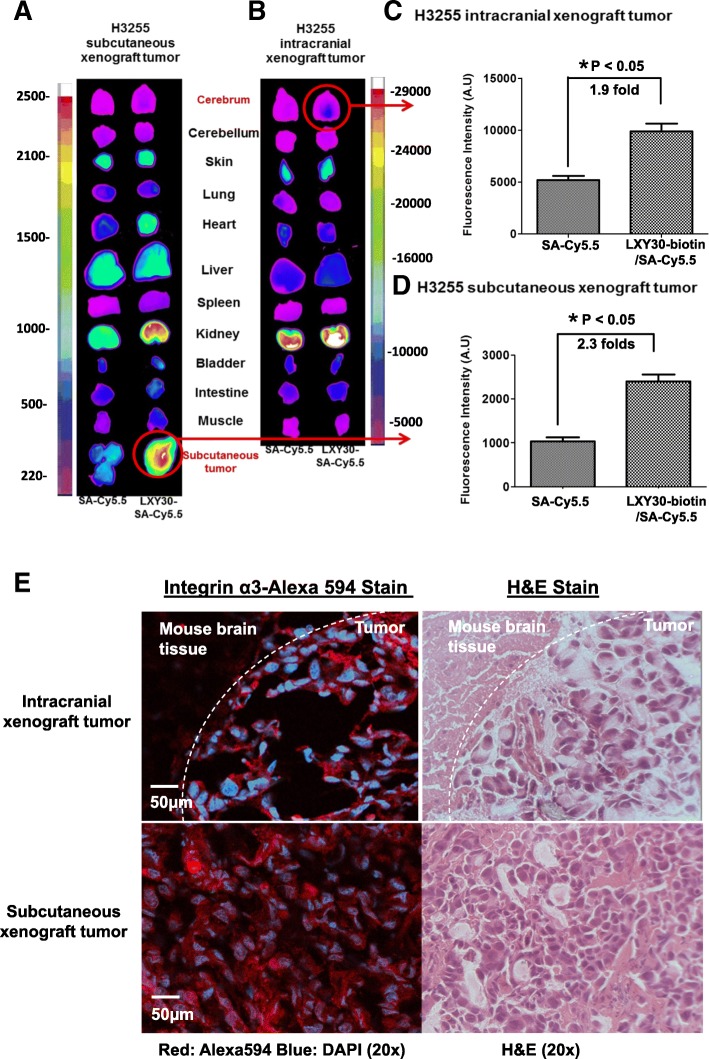
In vivo targeting of LXY30 to both subcutaneous and intracranial xenograft tumors of EGFR-mutant H3255 cells. Optical images were taken 6 h after the injection of streptavidin-Cy5.5 (SA-Cy5.5) alone or in combination with biotinylated LXY30 (LXY30-biotin) into nude mice bearing subcutaneous (**a**) or intracranial *EGFR-mutant* lung cancer H3255 xenografts (**b**). The fluorescence signals of SA-Cy5.5 and LXY30-SA-Cy5.5 were quantitated in both intracranial (**c**) and subcutaneous (**d**) H3255 xenograft tumors. Fluorescence microscopy image and H&E staining of intracranial and subcutaneous H3255 xenograft tumors (**e**), showing LXY30 only accumulated in H3255 xenograft tumor cells but not the surrounding normal brain tissue

**Fig. 7:**
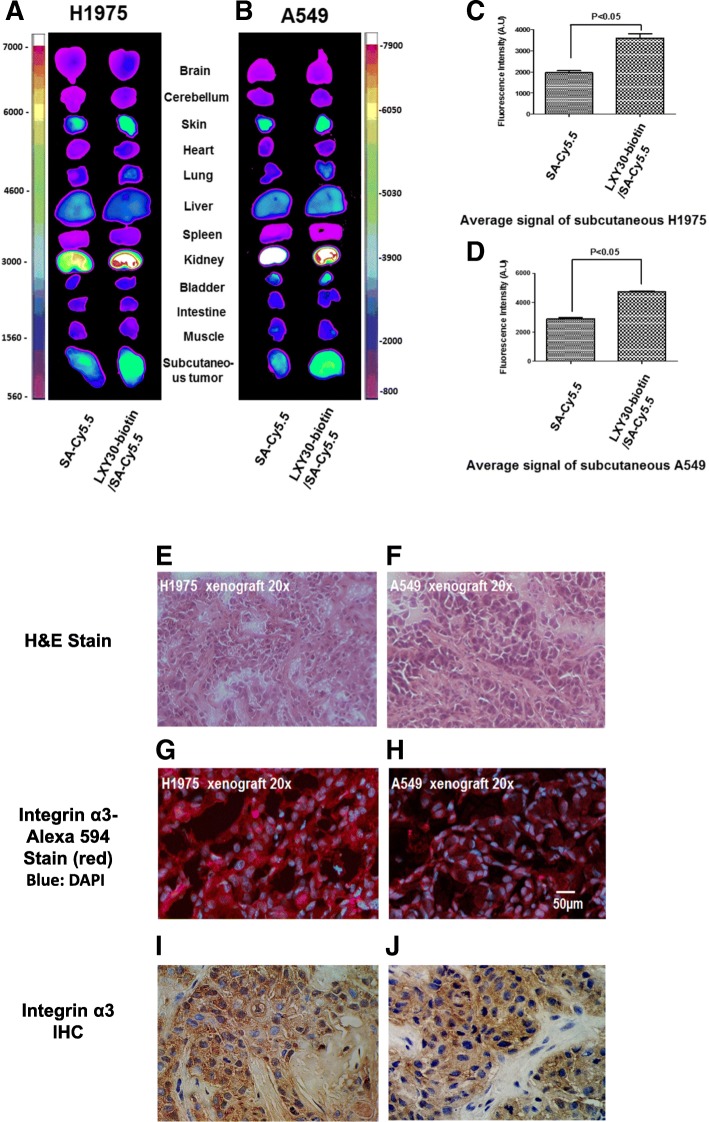
Optical images showing preferential uptakes of LXY30-biotin-streptavidin-Cy5.5 conjugate (i.e., drug surrogate) in EGFR-resistant lung cancer xenografts in nude mice. After conjugated with streptavidin-Cy5.5, LXY30 shows the capability to deliver imaging dye to subcutaneous xenografts of H1975 (**a**, **c**) and A549 (**b**, **d**). Representative images of tumor sections were stained by H&E staining (**e**, **f**), fluorescent (Alexa 594)-labeled anti-α3 integrin antibody (**g**, **h**), and integrin α3 IHC (**i**, **j**). *p < 0.05

The in vivo tumor-targeting effect of LXY30 was evaluated in a subcutaneous and intracranial mouse models generated from EGFR-mutant lung adenocarcinoma H3255 cells by optical imaging (Fig. 6). We found that LXY30-biotin/streptavidin-Cy5.5 dye complex was accumulated in the subcutaneous and intracranial xenografts of H3255 cells (Fig 6a, b, respectively). In both subcutaneous and intracranial xenografts, there was about twofold higher uptakes of SA-Cy5.5 dye with LXY30 complexed compared to those without LXY30 in the imaging complex (Fig. 6c, d, respectively). The expression of α3 integrin was detected by fluorescence stain in tumor cells but not surrounding normal tissues in subcutaneous and intracranial xenografts (Fig. 6e, left panel). The histopathology of subcutaneous and intracranial xenografts was confirmed by H&E stain (Fig. 6e, right panel). The in vivo targeting effect of LXY30 was also shown in subcutaneous xenograft models of H1975 (EGFR L858R/T790 M mutations) and A549 (KRAS G12S mutation) (Fig. 7).

### LXY30 specifically targets lung squamous cell cancer

**Fig. 8:**
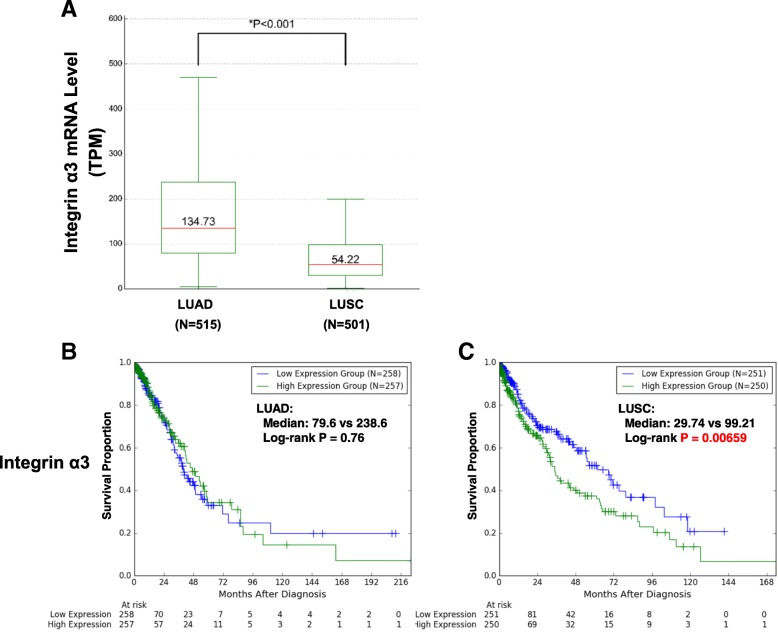
High α3 integrin expression level is associated with poor prognosis in NSCLC patients. The transcriptome expression data from lung cancer patients were analyzed in The Cancer Genome Atlas (TCGA) database (http://www.cancergenome.nih.gov) including 517 lung adenocarcinoma (LUAD) and 502 lung squamous cell carcinoma (LUSC) samples. **a** The RNA expression levels of α3 integrin (INTA3) gene in LUAD and LUSC, respectively. Kaplan-Meier curves of overall survival were stratified by ITGA3 expression in LUAD (**b**) and LUSC (**c**)

The above studies were performed in lung adenocarcinoma models. There are very few lung squamous cell models for research use. We analyzed the RNA levels of α3 integrin (INTA3) gene in 517 lung adenocarcinoma (LUAD) and 502 lung squamous cell carcinoma (LUSC) tumor samples using the transcriptome expression data from lung cancer patients in The Cancer Genome Atlas (TCGA) database (http://www.cancergenome.nih.gov). We found that the expression of α3 integrin levels was significantly higher in LUAD than in LUSC (134.73 vs 54.22, *p* < 0.001) (Fig. 8a). High ITGA3 expression was associated with poorer prognosis in LUSC (Fig. 8c) but not in LUAD (Fig. 8b).

**Fig. 9:**
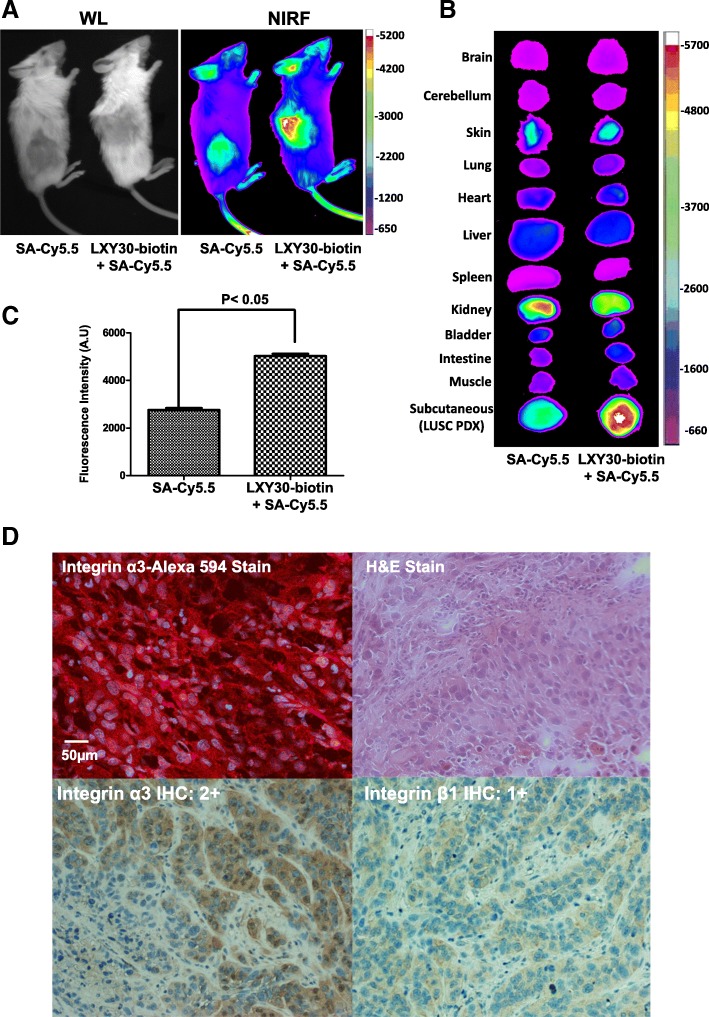
LXY30 specifically targets subcutaneous PDX xenograft tumors. A lung squamous cell (LUSC) PDX model was generated by engrafting biopsy specimen into NSG mice as described in the “Materials and methods” section. Optical images by white light (WL) and in vivo fluorescence images (**a**) were taken 6 h after the injection of streptavidin-Cy5.5 (SA-Cy5.5) either alone or together with biotinylated LXY30 (LXY30-biotin) into NSG mice bearing subcutaneous LUSC PDX xenografts. Major organs from the NSG mice were subjected to *ex vivo* imaging immediately after the mice were sacrificed (**b**). The fluorescence signals of SA-Cy5.5 and LXY30-Biotin-SA-Cy5.5 were further quantitated for subcutaneous LUSC PDX tumors (**c**). Representative images of LUSC PDX tumor sections were stained by fluorescent (Alexa594)-labeled anti-α3 integrin antibody, H&E, integrin α3, and integrin β1 immunohistochemistry stains (**d**). PDX, patient-derived xenograft; LUSC, lung squamous carcinoma; WL, white light

We thus tested the in vivo targeting effect of LXY30 on a PDX model that was generated from a patient with metastatic lung squamous cell cancer [28]. Histopathological assessment showed that the tumors were freshly formed without any treatment effect [28]. The LXY30-biotin-streptavidin (SA)-Cy5.5 dye complex had significantly increased uptake compared to SA-Cy5.5 dye complex in tumor xenografts as shown in in vivo (Fig. 9a) and ex vivo imaging (Fig. 9b, c). The expression of α3 integrin was confirmed by fluorescence stain (Fig 9d), similar to these xenografts derived from human lung adenocarcinoma cell lines (Fig. 6 and Fig. 7).

## Discussion

Several studies have shown that α3β1 integrin is weakly detected in the basement membranes of alveolar walls and is highly expressed in primary lung cancer cells [34–36]. Increased α3β1 integrin expression in tumor cells mediated tumor invasion and metastasis [14, 29, 37]. The key innovation of our study is that the small (4–9 amino acids in length) peptide or peptide-like ligands generated by our group were structurally optimized to have high affinity binding to specific integrins overexpressed on live tumor cells [38]. Compared to the natural integrin ligands that have low binding affinity to integrins, these integrin-specific peptide ligands contain l-amino acids, d-amino acids, cyclic structure, and organic moieties, rendering them resistant to proteolysis. This latter attribute is essential for clinical applications. Among all the peptide ligands that we have generated to date, LXY30 targeting α3(β1) integrin, LXW64 targeting αvβ3 integrin, and LLP2A targeting α4β1 integrin are the most potent ligands for the respective integrin and have a broad-spectrum binding to multiple epithelial tumor types including NSCLC. α3β1 integrin is one of the most common integrin subtypes expressed on tumor cells mediating metastasis and treatment resistance. We found that LXY30 could specifically and sensitively bind to various NSCLC cells and tumor-derived exosomes (Fig. 1) as well as circulating tumor cells in malignant pleural effusion from 80% of NSCLC patients (Fig. 3). The clinical application of multiplexed molecular biomarker assays has revolutionized cancer diagnosis and treatment, enabling the current era of precision cancer medicine [3, 39, 40]. Currently, tumor genomic profiling assays by NGS (such as FoundationOne CDx assay) require tumor cells to be present in at least 20% of cells in the cell block derived from pleural effusion or archived tumor specimens [41]. In a patient with less than 5% tumor cells present in malignant pleural effusion, LXY30 was able to enrich the malignant tumor cells to over 20% for successful detection of genomic alterations. Work is currently underway in our laboratory to test the clinical utility of tumor cell enrichment from the malignant biofluids by LXY30 in metastatic NSCLC patients, such that the success rate of tumor genomic testing can be improved.

Genotyping of plasma cell-free DNA (cfDNA) has received US FDA approval [42] and has been increasingly used to complement tissue-based genomic assays in precision oncology [40, 43]. However, the sensitivity of the current FDA-approved companion diagnostics using plasma ctDNA for EGFR T790 M is 70–82% with a specificity of ≥ 95% [14–16]. Nanosize EVs derived from tumor cells protect internal contents such as DNA, RNA, and miRNA, and lipids and proteins from plasma nucleases and proteases and physiological clearance. They may serve as an alternative to blood ctDNA for revealing dynamic tumor genomic changes and guide personalized cancer care [44]. Compared to the plasma cfDNA that is in 150–200 bp fragments and has a half-life of < 2 h, tumor-derived exosomes should yield higher concentration and longer fragment of nucleic acids (DNA, RNA, and miRNA). The challenge is to isolate these nanosize tumor-specific exosomes from the vast majority of non-tumor-derived exosomes in patient’s plasma for clinical tumor genomic testing. In this study, we showed the DNA isolated from the LXY30-enriched exosomes contains high amount of DNA that could be used for tumor genotyping. Using this approach, large volume of cell-free body fluids (plasma, pleural effusion, or pericardial effusion) can serve as an alternative or complimentary resource to plasma cfDNA for tumor genomic profiling and early diagnosis. Furthermore, the expression of specific integrin subtypes has been linked to the organotropic metastasis of epithelial tumors [29]. This knowledge of exosome-mediated metastasis independent of cell-mediated metastasis is important for understanding the mechanisms of metastasis and developing therapeutic strategies to eliminate metastasis.

The presence of gain-of-function somatic mutations in the tyrosine kinase domain of the epidermal growth factor receptor (EGFR*)* gene defines the first molecular subset of metastatic NSCLC patients whose tumors have 60–84% response rate to first-line EGFR tyrosine kinase inhibitors (TKIs; i.e., erlotinib, gefitinib, afatinib, osimertinib). These patients have a median progression-free survival of 9–18 months and a median overall survival of 18–36 months [45–47]. The development of acquire resistance to EGFR TKIs is enviable. New strategies are needed to prevent and treat for the resistance to EGFR-targeting therapy. Approximately 50–60% of *EGFR*-mutant NSCLC patients were found to develop brain metastasis during their disease course [48]. Consistent with the report that the expression of α3β1 integrin was increased in erlotinib-resistant NSCLC tumors [49], we found that LXY30 was the most prevalent and potent integrin ligand for binding to live *EGFR-mutant* lung cancer. *EGFR*-mutant H3255 cells could alter their integrin expression profile during disease progression. Thus, longitudinal evaluation is needed for selecting appropriate integrin targets for individual lung cancer patients. LXY30 activates the EGFR signaling pathway via different downstream signaling molecules independently from other integrins such as αvβ3 integrin. Although α3β1 integrin can express in many normal organs and tissues in the humans and mice, LXY30 was found to preferentially target subcutaneous and intracranial tumors without binding to normal organs or surrounding normal brain tissues in vivo. This suggests that α3β1 integrin on tumor cells is overexpressed and/or has higher affinity to LXY30 than normal cells. We found that the LXY30/streptavidin complex could enter into the brain through the compromised BBB to tumor cells in mice bearing intracranial xenograft tumors, but was not able to cross the normal intact BBB in mice bearing subcutaneous xenograft only. LXY30 could also target PDX of lung squamous cell carcinoma. Together, our data indicate that LXY30 is an excellent probe to guide imaging agents and therapeutics to both intracranial and extracranial tumors.

Our study has several translational potentials. First, the detection of α3β1 integrin-expressing tumor cells and/or tumor-derived exosomes by LXY30 in the biofluids from patients with NSCLC suggests poor prognosis and tumor metastasis. Second, LXY30 could be used for sensitive detection of metastatic tumors or enrichment of the tumor cells in patient’s biofluids, and thus potentially increase the success rate on the molecular diagnosis of NSCLC. Third, the internalization property of LXY30 by α3β1 integrin-expressing cancer cells could be used to facilitate the delivery of conventional chemotherapeutic agents, target-specific agents, siRNAs, and microRNAs into tumor cells, either through direct conjugation or by encapsulation inside LXY30-decorated nanocarriers. Finally, the fact that LXY30-biotin/streptavidin-Cy5.5 complex with over 80,000 Da can target intracranially implanted xenografts suggests that LXY30 is an excellent cancer-specific ligand for guiding in vivo drug delivery to metastatic tumors in the brain.

## Conclusions

To the best of our knowledge, this is the first report demonstrating that a novel, potent integrin binding peptide LXY30 can detect and enrich live, circulating tumor cells and tumor-derived exosomes from human NSCLC cell lines and biofluids from patients with advanced NSCLC. LXY30 can modulate the activity of the EGFR signaling pathway. LXY30-biotin/streptavidin-Cy5.5 conjugate had preferentially higher uptakes in the subcutaneous and intracranial xenografts of various α3β1 integrin-expressing NSCLC and patient-derived NSCLC xenograft models compared to the surrounding normal tissues. Further studies are warranted to use LXY30 to increase the sensitivity of cancer detection, molecular diagnosis, and in vivo targeted delivery of imaging dye and cancer drugs in α3β1 integrin-expression NSCLC.

## Additional files

Additional file 1: **Figure S1.** (PPTX 79 kb)

Additional file 2: **Figure S2.** (PPTX 1330 kb)

## Data Availability

The dataset supporting the conclusions of this article is included within the article.

## Abbreviations

AKT: Protein kinase B
cfDNA: Cell-free DNA
CSPG4: Chondroitin sulfate proteoglycan 4
DLS: Dynamic light scattering
EGFR: Epidermal growth factor receptor
EV: Extracellular vesicles
FGFR: Fibroblast growth factor receptor
FITC: Fluorescein isothiocyanate
IRB: Institutional review board
ISEV: International Society for Extracellular Vesicles
LUAD: Lung adenocarcinoma
LUSC: Lung squamous carcinoma
MALDI-TOF MS: Matrix-assisted laser desorption/ionization time of flight mass spectrometry
NFκB: Nuclear factor kappa-light-chain-enhancer of activated B cells
NGS: Next-generation sequencing
NSCLC: Non-small cell lung carcinoma
NTA: Nanoparticle tracking analysis
O/N: Overnight
PBMCs: Pleural blood mononuclear cells
PDX: Patient-derived xenograft
PI3K: Phosphatidylinositol-4,5-bisphosphate 3-kinase
RTKs: Receptor tyrosine kinases
SD: Standard deviation
TCGA: The Cancer Genome Atlas
TEM: Transmission electron microscopy
WL: White light
