# Correction to: High-affinity peptide ligand LXY30 for targeting α3β1 integrin in non-small cell lung cancer

**DOI:** 10.1186/s13045-019-0764-z

**Published:** 2019-07-26

**Authors:** Wenwu Xiao, Weijie Ma, Sixi Wei, Qianping Li, Ruiwu Liu, Randy P. Carney, Kevin Yang, Joyce Lee, Alan Nyugen, Ken Y. Yoneda, Kit S. Lam, Tianhong Li

**Affiliations:** 1 Department of Biochemistry and Molecular Medicine, University of California Davis, Sacramento, CA 95817 USA; 2 Division of Hematology/Oncology, Department of Internal Medicine, University of California Davis School of Medicine, University of California Davis Comprehensive Cancer Center, 4501 X Street, Suite 3016, Sacramento, CA 95817 USA; 3 Present Address: Department of Biochemistry, Hospital Affiliated to Guizhou Medical University, Guiyang, Guizhou China; 4 Present Address: Department of Cardiothoracic Surgery, Shanghai Jiaotong University Affiliated Sixth People’s Hospital, 600 Yi-Shan Road, Shanghai, 200233 China; 5 Department of Biomedical Engineering, University of California Davis, Davis, CA USA; 6 Present Address: Perelman School of Medicine, University of Pennsylvania, Philadelphia, PA USA; 7 Department of Pharmacy, University of California Davis Health System, Sacramento, CA 95817 USA; 8 Division of Pulmonary, Critical Care, and Sleep Medicine, Department of Internal Medicine, University of California Davis School of Medicine, Sacramento, CA USA; 9 Department of Internal Medicine, Veterans Affairs Northern California Health Care System, Mather, CA USA

**Correction to: J Hematol Oncol (2019) 12:56**

**https://doi.org/10.1186/s13045-019-0740-7**

The original article [1] contains an error in Fig. 2 whereby Fig. 2d has mistakenly been omitted. Figure 2 can be viewed in its entirety – including Fig. 2d – in this Correction article.

**Fig. 2:**
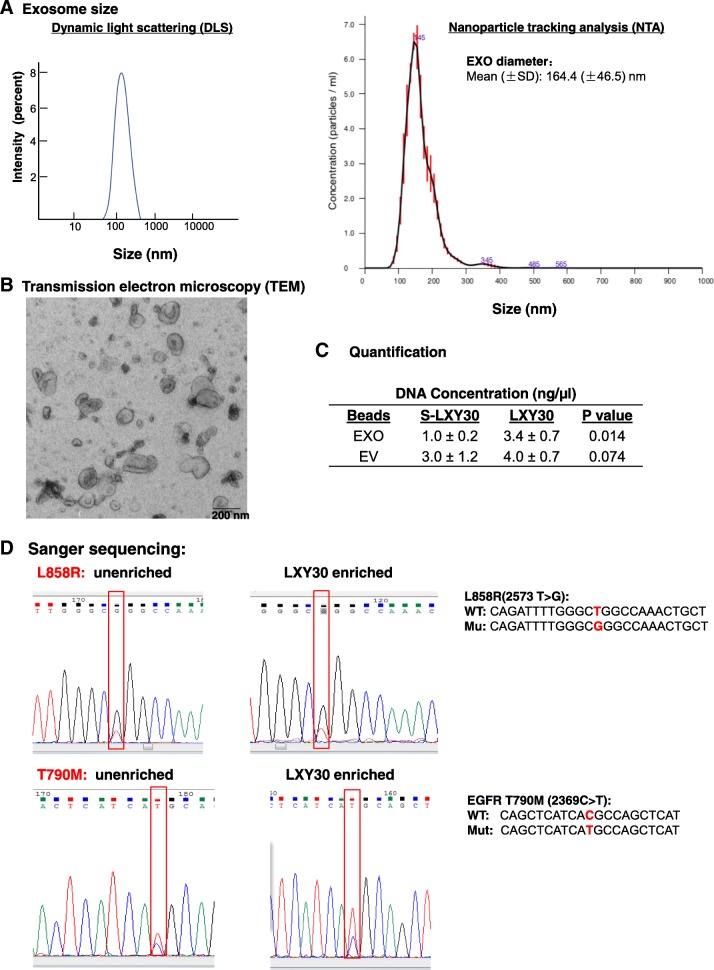
Characterization of tumor derived exosomes and EVs. The size and morphology of exosomes were evaluated by dynamic light scattering (DLS) and nanoparticle tracking analysis (NTA) (**a**) and transmission electron microscopy (TEM) (**b**), respectively. The yield of DNA was 3.4-fold higher in LXY30-enriched exosomes than in S-LXY30-enriched exosomes (3.4 ± 0.7 vs 1.0 ± 0.2 ng/μL, *p* = 0.014) (**c**). Driven mutations (EGFR L858R and T790 M in H1975) were detected by PCR and Sanger sequencing in the DNA isolated from LXY30 exosomes (**d**)
