# Supplementary figures and images for: High-affinity peptide ligand LXY30 for targeting α3β1 integrin in non-small cell lung cancer
Source: J Hematol Oncol. 2019 Jun 10;12:56. doi: 10.1186/s13045-019-0740-7 (PMC6558829; doi:10.1186/s13045-019-0740-7)

## Slide 1
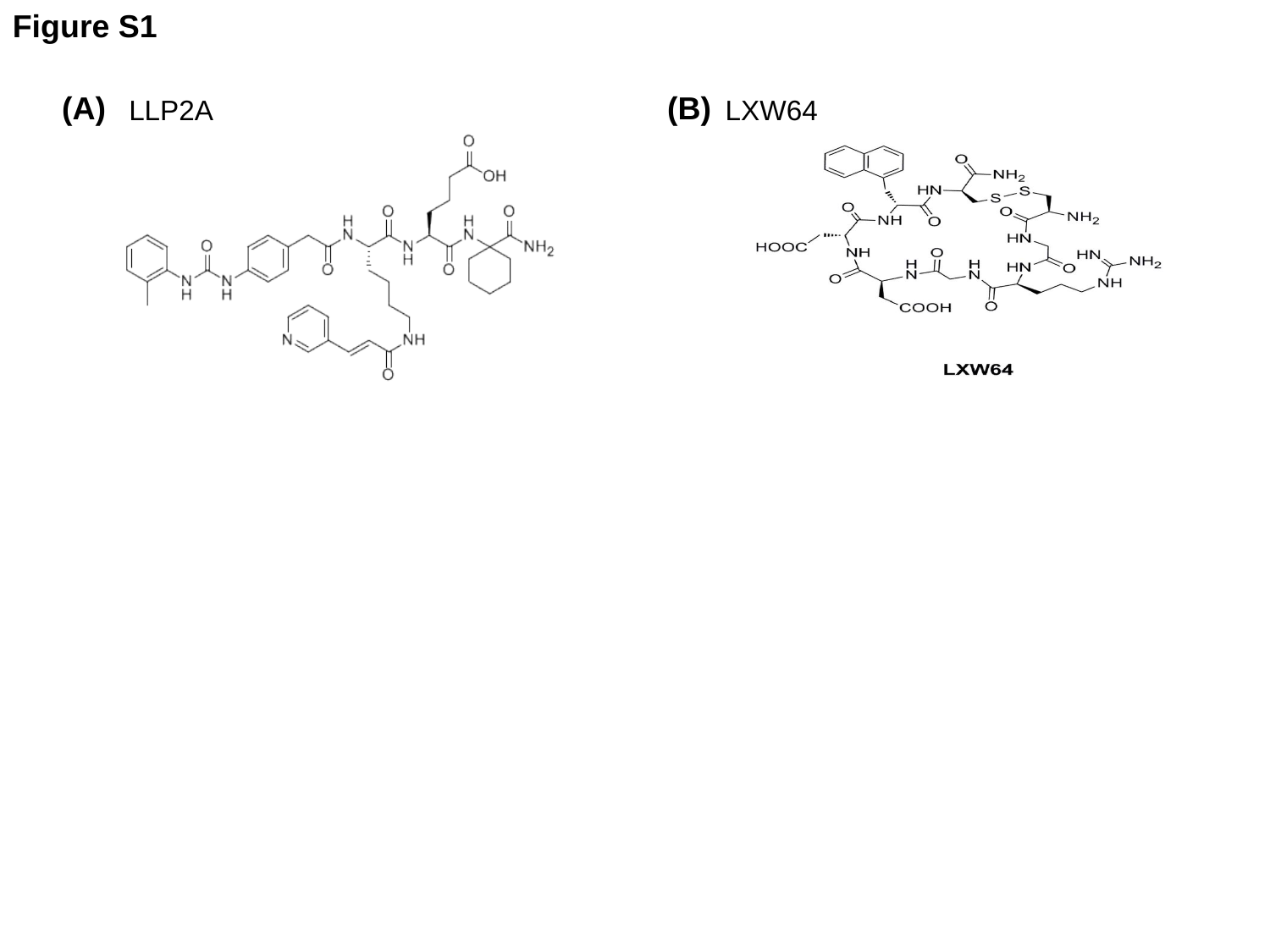

Figure S1
(A)				 (B)
LLP2A
LXW64

Supplement: Supplementary file 1 — Figure S1. (PPTX 79 kb) [file 13045_2019_740_MOESM1_ESM.pptx]
