# Supplementary material for: High-affinity peptide ligand LXY30 for targeting α3β1 integrin in non-small cell lung cancer
Source: J Hematol Oncol. 2019 Jun 10;12:56. doi: 10.1186/s13045-019-0740-7 (PMC6558829; doi:10.1186/s13045-019-0740-7)
Supplement: Supplementary file 2 — Figure S2. (PPTX 1330 kb) [file 13045_2019_740_MOESM2_ESM.pptx]

## Slide 1
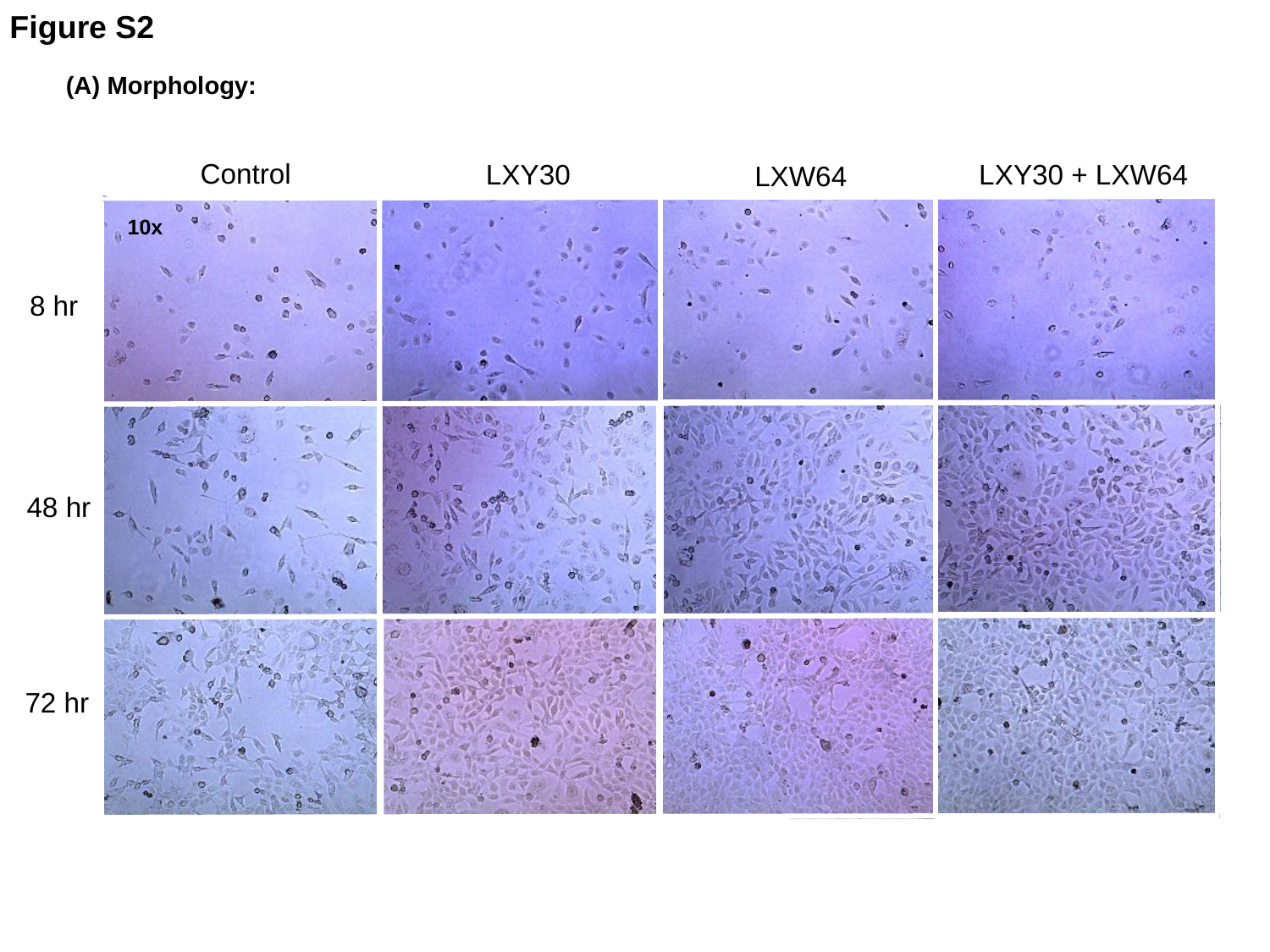

Figure S2
(A) Morphology:
Control
LXY30
LXY30 + LXW64
LXW64
10x
8 hr
48 hr
10x
10x
72 hr

## Slide 2
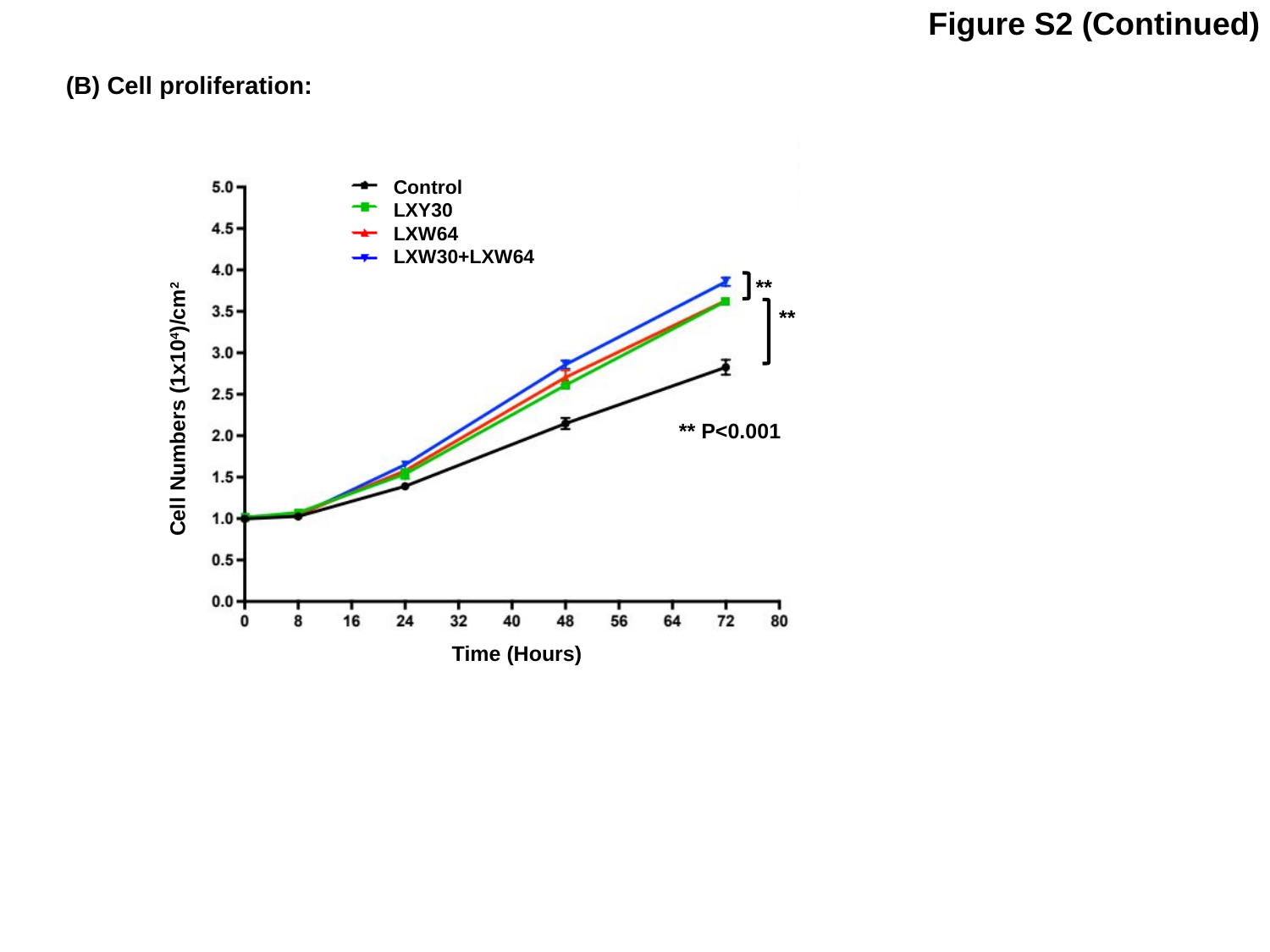

Figure S2 (Continued)
(B) Cell proliferation:
Control
LXY30
LXW64
LXW30+LXW64
**
**
Cell Numbers (1x10^4^)/cm2
Time (Hours)
** P<0.001
